# Assessment of Chocolate, Cheese, and Caffeine as Migraine Triggers: A Systematic Review and Meta‐Analysis of Patient‐Reported Trigger Rates

**DOI:** 10.1155/jnme/4693275

**Published:** 2026-08-26

**Authors:** Hayat Alzahrani

**Affiliations:** ^1^ Department of Food Sciences and Nutrition, College of Food and Agriculture Sciences, King Saud University, Riyadh, Saudi Arabia, ksu.edu.sa

**Keywords:** caffeine, cheese, chocolate, meta-analysis, migraine, systematic review

## Abstract

**Background:**

Migraine is a common neurological disorder affecting approximately 14%–15% of the global population and is a leading cause of years lived with disability. Dietary factors, including chocolate, caffeine, and cheese, are frequently reported as migraine triggers; however, their prevalence and contribution to migraine attacks remain inconsistent across studies. This systematic review and meta‐analysis aimed to estimate the pooled prevalence of chocolate, caffeine, and cheese as patient‐reported migraine triggers.

**Methods:**

This systematic review and meta‐analysis was conducted in accordance with the Cochrane Handbook for Systematic Reviews of Interventions and reported following the PRISMA 2020 Statement and MOOSE guidelines. A systematic search of PubMed, MEDLINE (via Ovid), Ovid, and Scopus was conducted from database inception to November 11, 2024. Observational studies reporting quantitative data on chocolate, caffeine, or cheese as migraine triggers were included. Data were synthesized using a random‐effects meta‐analysis of proportions in Jamovi (Version 2.3).

**Results:**

Eight studies included, but seven only contributed to the quantitative meta‐analysis. The pooled prevalence of patient‐reported migraine triggers was 13.9% for chocolate (95% CI: 7.0%–21.0%), 8.0% for cheese (95% CI: 3.0%–12.0%), and 12.0% for caffeine (95% CI: 6.0%–19.0%) (all *p* < 0.001). Substantial heterogeneity was observed across all analyses (I^2^ > 95%). Four studies were rated as good quality and four as fair quality using the NHLBI Quality Assessment Tool.

**Conclusions:**

Chocolate, caffeine, and cheese are commonly reported migraine triggers; however, their effects vary considerably among individuals. Given the substantial heterogeneity and reliance on observational, self‐reported data, routine avoidance of these foods cannot be universally recommended. Instead, individualized trigger identification, supported by headache diaries, should guide dietary management. Further well‐designed prospective studies are needed to clarify causal relationships.

## 1. Introduction

Migraine is a primary headache disorder characterized by recurrent headaches, often unilateral, pulsating, and of moderate‐to‐severe intensity. These headaches are accompanied by nausea, vomiting, and sensitivity to light and sound [1]. Migraines can be classified into episodic and chronic types, with chronic migraine being defined as headaches occurring on 15 or more days per month for over three months, with migraine features on at least eight days per month [2]. The global migraine prevalence is 14%–15%, and it accounts for up to 5% of global ill health, quantified as years lived with disability [3]. These clinical and epidemiological characteristics highlight migraine as a highly prevalent and disabling neurological disorder with significant public health implications.

Migraines have imposed a significant burden across physical, psychological, and economic domains. Migraines lead to frequent pain and disability, limiting daily activities and productivity. In addition, migraine attacks are linked to mental health issues like anxiety and depression, and the fear of future attacks, which can cause isolation and distress. As well, it has an economic burden as migraine generates high costs, including direct medical expenses and productivity losses, affecting both individuals and society [4]. Given this substantial burden, understanding modifiable factors that contribute to migraine is essential for improving patient outcomes.

Migraine management includes both acute and preventive pharmacological therapies. Acute treatment commonly involves nonsteroidal anti‐inflammatory drugs (NSAIDs), acetaminophen, triptans, ditans, and gepants, while preventive options include *β*‐blockers, antidepressants, antiepileptic drugs, calcium channel blockers, onabotulinumtoxinA for chronic migraine, and calcitonin gene‐related peptide (CGRP)–targeted therapies, including monoclonal antibodies and oral CGRP receptor antagonists [5–7]. Despite these therapeutic advances, many patients continue to experience recurrent attacks or treatment limitations, underscoring the importance of identifying modifiable factors, including dietary triggers, that may improve migraine prevention and overall disease management [8].

It is known that a range of migraine triggers includes environmental, lifestyle, and dietary factors. Environmental triggers include weather changes, bright lights, loud noises, and strong smells, with hormonal fluctuations also affecting susceptibility. Lifestyle factors such as stress, poor sleep quality, irregular sleep patterns, and high levels of physical exertion are common triggers, as are sudden changes in daily routines [9]. Additionally, certain foods and drinks, including caffeine, aged cheeses, and items containing additives like MSG or aspartame, can provoke migraines in some individuals. Fasting or skipping meals may also contribute to migraine onset. Among these, dietary factors are of particular interest because they are potentially modifiable and may offer an important target for prevention. Recognizing and managing these diverse triggers may help reduce the frequency and intensity of migraines [10].

Certain foods may provoke migraines by influencing blood vessel dilation, neurotransmitter levels, or inflammatory pathways. Common dietary triggers include caffeine, chocolate, and aged cheeses, all of which contain compounds like tyramine or phenylethylamine that may affect blood flow or neural activity [11]. Sudden changes in blood sugar due to consuming high‐sugar foods can trigger migraines by impairing metabolic stability [12]. Another study found that chocolate may influence migraines by affecting serotonin and vascular regulation, though its trigger role varies among individuals. In addition, excessive or irregular caffeine consumption, as well as caffeine withdrawal, can trigger migraines in susceptible individuals. Aged cheeses that have high tyramine might provoke migraines due to their impact on blood vessel function and neurotransmitter activity [13]. However, despite these proposed mechanisms, the relationship between specific dietary components and migraine remains inconsistent across individuals and studies. Thus, identifying and avoiding these food‐related triggers can be beneficial for migraine management and prevention.

Importantly, while many studies have explored individual dietary triggers, there is variability in the reported prevalence and impact of these triggers across populations. A previous systematic review has summarized the available evidence and identified chocolate, caffeine, alcohol, fasting, and certain dairy products among the most commonly reported dietary triggers [14]. However, this review was qualitative in nature and highlighted substantial heterogeneity and limited‐quality evidence across studies. To our knowledge, no recent meta‐analysis has comprehensively pooled the prevalence of individual dietary triggers among people with migraine. Therefore, this review aims to address this gap by systematically evaluating the available evidence and providing an updated quantitative synthesis to support dietary recommendations for migraine management.

## 2. Methods

This systematic review and meta‐analysis was conducted in accordance with the Cochrane Handbook for Systematic Reviews of Interventions and reported in accordance with the Preferred Reporting Items for Systematic Reviews and Meta‐Analyses (PRISMA) 2020 Statement and the Meta‐analysis of Observational Studies in Epidemiology (MOOSE) reporting guidelines [15–17].

### 2.1. Eligibility Criteria

#### 2.1.1. Type of the Included Studies

Observational studies (including cross‐sectional, prospective, retrospective, and observational case series).

#### 2.1.2. Participants

Adults or children diagnosed with primary headaches or migraines.

#### 2.1.3. Exposures

Studies examining the role of chocolate, caffeine, and/or cheese as triggers for migraine attacks.

#### 2.1.4. Outcomes

The trigger rates of chocolate, cheese, and caffeine as causes of migraines among the participants.

#### 2.1.5. Exclusion Criteria

Studies were excluded if they were not original research (e.g., reviews, editorials); focused only on nondietary triggers for migraines (e.g., stress, environmental factors); and did not provide quantitative data on the association between chocolate, caffeine, or cheese and migraines.

#### 2.1.6. Search Strategy

The search strategy was designed to ensure a comprehensive and reproducible identification of relevant literature. A systematic search was conducted across four major electronic databases: PubMed, MEDLINE (accessed via Ovid), Ovid, and Scopus. These databases were selected because they collectively provide extensive coverage of biomedical, clinical, and multidisciplinary research, thereby minimizing the risk of missing relevant studies.

No language or publication‐type filters were applied to reduce selection bias and capture the full breadth of available evidence. The search included all studies published from database inception up to November 11, 2024.

A combination of controlled vocabulary (MeSH terms) and relevant keywords was used to improve search sensitivity and specificity. The following MeSH terms and keywords were included: “Migraine,” “dietary triggers,” “cheese,” “chocolate,” and “caffeine.” These terms were selected to capture literature investigating common dietary factors associated with migraine onset. Boolean operators (AND, OR) were applied to appropriately combine terms and refine the search results.

#### 2.1.7. Selection of Studies

The processes of online search, screening the titles and abstracts, as well as revising the full text of relevant articles, were conducted by one independent researcher.

#### 2.1.8. Data Extraction

Data extracted included the following data: (1) authors’ details; (2) characteristics of the population (including age and the sample size); (3) the specific association between chocolate, caffeine, and cheese and migraine onset or frequence; (4) rates of chocolate, caffeine, and cheese as migraine triggers, effect sizes, or odds ratios, as reported by the studies.

### 2.2. Assessment of the Quality of the Included Studies

The methodological quality of the included observational studies was independently assessed using the National Heart, Lung, and Blood Institute (NHLBI) Quality Assessment Tool for Observational Cohort and Cross‐Sectional Studies. This tool evaluates key methodological domains, including the clarity of the research question, definition of the study population, participation rate, sample size justification, assessment of exposures and outcomes, measurement reliability, adjustment for potential confounding variables, and appropriateness of the statistical analyses. Each study was rated as good, fair, or poor according to the NHLBI guidance [18].

### 2.3. Data Synthesis

Meta‐analysis was performed using Jamovi (Version 2.3) [19]. Heterogeneity was evaluated using Cochrane’s chi‐squared test, with a significance threshold set at 0.10, and the I2 statistic, where a value greater than 50% indicated significant heterogeneity [20]. Only studies reporting sufficient numerical data were included in the quantitative meta‐analysis. Studies lacking extractable outcome data were included in the qualitative synthesis only. Owing to the anticipated clinical and methodological variability among the included studies, a random‐effects (RE) model with the restricted maximum likelihood (REML) estimator was used. To address the high statistical heterogeneity (I2 > 95%) observed across the primary outcomes, sensitivity analyses were performed using the “leave‐one‐out” method to identify potential outlier studies that disproportionately influenced the pooled estimates. The outcome of interest was the rates of chocolate, caffeine, and cheese as triggers for migraines across the included studies. Thus, meta‐analysis of proportions was adopted to calculate the pooled proportion of migraine triggers. Statistical significance was set at a *p*‐value of less than 0.05. Publication bias was planned to be assessed using funnel plots and Egger’s regression test when at least 10 studies were available for a meta‐analysis. However, because fewer than 10 studies were included, publication bias was not formally evaluated, in accordance with the recommendations of the Cochrane Handbook for Systematic Reviews of Interventions [21].

## 3. Results

### 3.1. Results of the Literature Search and Study Selection

**FIGURE 1:**
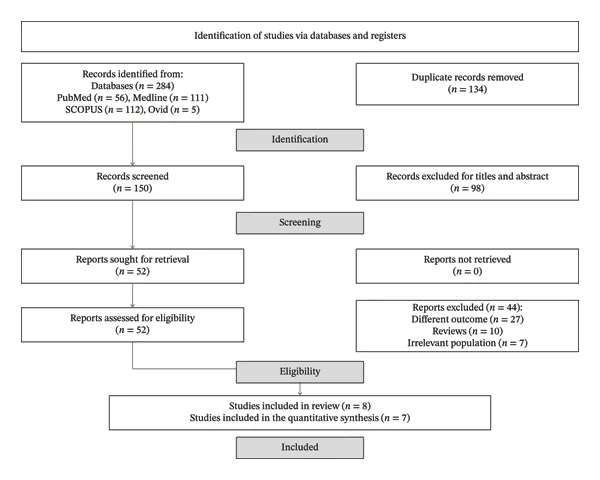
PRISMA flowchart selection process.

The search strategy yielded 284 records, of which 134 duplicates were removed. The remaining 150 records underwent screening of their titles and abstracts, and 98 records were excluded. The remaining 52 records were retrieved for full‐text screening and were assessed for eligibility. Of these, 44 records were excluded. Finally, eight studies were eligible for inclusion in the present review [22–29]. Of these, seven provided sufficient numerical data for inclusion in the quantitative meta‐analysis. One study [23] was included in the qualitative synthesis only because it did not report sufficient numerical data to permit quantitative pooling. Figure 1 illustrates the details of the screening process in the PRISMA flowchart (Figure 1).

#### 3.1.1. Characteristics of the Included Studies

The systematic review included eight studies that focused on headache diaries and migraine triggers. Participants in the studies record headache characteristics and potential triggers over a certain period. Studies were conducted in countries like the United States [27, 28], the United Kingdom [24], Austria [23], Turkey [26], Brazil [29], South Korea [25], and Malaysia [22]. The study designs were primarily survey‐based or observational. Andress‐Rothrock [28], Fukui [29], and Mollaoglu [26] utilized survey designs, where participants completed questionnaires about migraine triggers. Park [25] implemented an electronic headache diary design using a smartphone app to track triggers in real time. In addition, Peris [23] conducted a retrospective analysis, and Schürks [27] conducted a cross‐sectional observational study. Taheri [24] and Tai [22] employed prospective observational designs, with Taheri conducting a case series on dietary changes in children and Tai utilizing a cross‐sectional survey to assess triggers among patients. Among these studies, participants were primarily adult patients with a higher representation of females. Most studies included patients from specific clinics or used apps to track triggers, while sample sizes ranged from small groups of 62 to larger populations of over 9000. The mean ages of participants varied around the late 30s to early 40s, except for one study focused on children (mean age 10.5 years) [24]. The reported headache and migraine triggers differed among the included studies. Chocolate, cheese, and caffeine emerged as common dietary triggers (Table 1).

**TABLE 1:** Characteristics of the included studies.

Study ID	Country	Aim	Participants	Migraine characteristic	Study design	Data collection tool	Methodology	Findings
Tai ML et al, 2018 [22]	Malaysia	To examine the dietary trigger factors of migraine and tension‐type headache (TTH) among Malaysian patients of different ethnic groups (Malay, Chinese, and Indian)	684 patients (319 with migraine, 365 with TTH)	Included migraine with aura, migraine without aura, and chronic migraine.	Prospective cross‐sectional study.	Structured headache questionnaire with a checklist of 25 dietary triggers, along with demographic data collection.	Univariate and logistic regression analyses were conducted to assess the association of dietary triggers with migraine and TTH.	37.3% reported dietary triggers, with coffee (19.9%), chocolate (7.5%), and monosodium glutamate (MSG) (5.6%) being the most common. Chocolate (OR 2.16, *p* = 0.035) and coffee (OR 1.73, *p* = 0.014) were significantly associated with migraine compared to TTH.

Peris F et al, 2017 [23] ^∗^	Austria	Identify potential trigger factor‐attack associations for migraine in individual patients and examine inter‐individual variability in trigger profiles.	326 migraine patients (85.3% female)	Migraine with or without aura; average of 2.9 attacks per month per patient; factors potentially linked to migraines tracked over 90 days.	Retrospective analysis of patient‐reported data from the PAMINA study.	Paper diaries documenting 33 potential migraine‐related factors daily for 90 days.	*N* = 1 statistical analysis of individual factor‐attack associations using Cox proportional hazards models	87% of patients had individual trigger profiles, averaging four significant factors. Common triggers included neck tension, tiredness, sleep issues, etc. Chocolate was included as one of the 33 factors analyzed, but was found to be a less common trigger. It was reported as a significant factor for migraines in less than 10% of patients, indicating that it is not a prominent trigger across the study population.

Taheri S, 2017 [24]	United Kingdom	To evaluate the effect of excluding frequently consumed dietary triggers in children with chronic primary headaches.	115 children (48 females, 67 males), ages 3–15 years (mean age 10.5 years)	Primary headache types included migraine without aura (57 patients), migraine with aura (2), tension‐type headaches, and abdominal migraines.	Prospective observational case series	Food and headache diaries were used to document headache frequency, severity, and dietary intake during a 6‐week exclusion period	Patients eliminated suspected food triggers for 6 weeks; follow‐ups assessed headache frequency and severity reduction with food exclusions	87% of patients saw complete headache resolution with the exclusion of 1–3 foods. Common triggers: caffeine (28%), MSG (25%), cocoa (22%), cheese (13%).

Park JW et al, 2016 [25]	South Korea	To analyze trigger factors for episodic migraines using a smartphone headache diary application (SHD) to improve understanding of migraine occurrences.	62 episodic migraine patients (mean age 37.7 years, 82.3% female), experiencing 2–14 headache days per month.	1099 headache days recorded: 336 migraines (30.6%) and 763 non‐migraine headaches.	Prospective observational study using a smartphone application over 3 months.	Smartphone Headache Diary (SHD) application to record daily triggers and headache details.	Participants recorded daily headache occurrences, trigger exposure, intensity, duration, and associated factors. Statistical analysis included logistic regression.	Key triggers included stress (57.7% likelihood), sleep deprivation (55.1%), and fatigue (48.5%). Common migraine triggers were hormonal changes, noise, and alcohol, with higher severity and disability when triggers were present. Chocolate and Cheese were relatively uncommon as migraine triggers, appearing in only 1.5% of migraine cases. Although they were tracked, they were not significant factors for most participants. Caffeine was also a less frequent trigger, with 2.4% of migraine cases associated with caffeine intake. It did not significantly influence migraine occurrences across the study population.

Mollaoğlu M, 2013 [26]	Turkey	To investigate trigger factors for migraines in patients.	126 patients (86 females and 40 males); mean age: 36.32	Included patients with migraine with aura (MA) and without aura (MwA)	Cross‐sectional study conducted at Cumhuriyet University Hospital, Sivas, Turkey.	Personal Information Form (PIF) and List of Trigger Factors of Migraine (LTFM), which included Likert‐scale questions on triggers such as dietary, hormonal, sleep, stress, and environmental factors.	Statistical analyses were performed with chi‐square tests to explore relationships between migraine types and triggers.	**Chocolate**: Reported as a dietary trigger by 18.3% of participants. Cheese: A trigger for 10.3% of patients. **Caffeine:** Coffee triggered migraines in 6.3% of patients. Other significant triggers included stress (78.6%), sleep disturbances (64.2%), and environmental factors (26.1%).

Schürks M et al, 2011[27]	USA	To identify components of migraine characteristics by grouping information on features, symptoms, and triggers within a large cohort of women from the Women’s Health Study (WHS).	65,169 U.S. female health professionals, aged 45 and older	Among the 1675 women in the subgroup with detailed migraine information, common symptoms included sensitivity to light (93%) and nausea/vomiting (89.1%). Migraine frequency was high, with 52.6% of participants reporting monthly attacks, and 57.8% indicating that migraines interfered with daily functioning.	Cross‐sectional observational study	The baseline questionnaire collected data on migraine history, including attack frequency, location, severity, and associated symptoms (e.g., nausea, sensitivity to light and sound). The detailed migraine‐specific questionnaire gathered additional information on potential migraine triggers (such as food and physical activity) and migraine‐related symptoms.	The study used principal component analysis (PCA), a statistical technique that simplifies complex data sets by identifying main components (eigenvalues > 1), summarizing multiple variables.	The PCA revealed distinct components that capture the migraine experience: Five Primary Components in the detailed subgroup (*n* = 1675): food triggers, nonspecific symptoms, CNS sensitization, migraine severity, and aura/visual symptoms. The foods included were: • Cheeses • Chocolate • Artificial sweeteners • Cold cuts or preserved meats • Monosodium glutamate (MSG) • Red wine • White wine • Other alcohol • Coffee • Tea • Cola drinks

Andress‐Rothrock D et al., 2010 [28]	USA	To analyze the prevalence and types of migraine triggers in a clinic‐based population and assess any influences of gender, migraine subtype, or duration on trigger prevalence	200 consecutive new migraine patients (172 females, 143 Caucasians, 55 African Americans)	Participants included patients with episodic migraine (EM) and chronic migraine (CM) diagnosed according to the International Classification of Headache Disorders (ICHD‐II)	Observational clinic‐based study	Intake questionnaire covering demographic details, headache history, and a checklist of specific migraine triggers	Descriptive analysis was conducted, and no statistical tests were performed to assess differences	91% of patients reported at least one trigger, with emotional stress (59%), sleep disruptions (53.5%), odors (46.5%), and missing meals (39%) as the most common. Specifically, **3% reported chocolate, 3% cheese, and 8% caffeine as migraine triggers.**

Fukui PT et al, 2008 [29]	Brazil	To analyze precipitating (trigger) factors in a sample of migraine patients	200 migraine patients (162 women and 38 men)	Migraine patients diagnosed according to the International Classification of Headache Disorders	Observational study using structured interviews with a predetermined list of common migraine triggers	Structured interviews with a predetermined list of potential trigger factors	The study employed chi‐square tests to analyze the significance of trigger factors among patients, including a comparison of responses between genders.	84.5% reported dietary triggers (fasting most common): ‐ Chocolate: Reported as a trigger by 20.5% of participants (22.8% of women and 10.5% of men). ‐ Cheese: Reported as a trigger by 8.5% of participants (9.3% of women and 5.3% of men). ‐ Caffeine: Reported as a trigger by 14.5% of participants, with coffee being the specific source. This included 13% of women and 21.1% of men

^∗^Included in the qualitative synthesis only because sufficient numerical data were unavailable for quantitative meta‐analysis.

#### 3.1.2. Chocolate Trigger Rate

**FIGURE 2:**
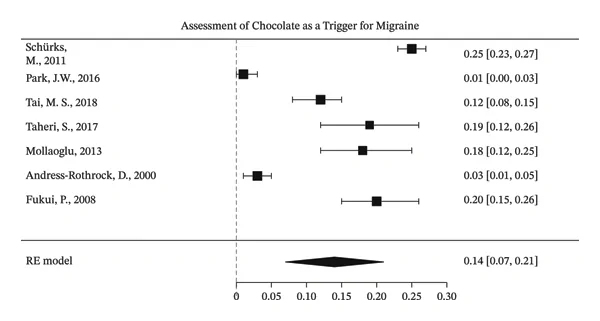
Assessment of chocolate as a trigger for migraine (*n* = 7). Peris et al. were not included because extractable numerical data were unavailable.

The RE model, incorporating data from seven studies, estimates a 13.9% trigger rate for chocolate‐induced migraines. The pooled estimate from the RE model was 0.14 (95% confidence interval [CI]: 0.07 to 0.21, *p* < 0.001). This shows that chocolate may trigger migraines in approximately 14% of cases. Heterogeneity among the studies was substantial, as indicated by an I2 value of 97.57%. This high level of heterogeneity suggests significant variability in the findings, attributable to differences in study design, population characteristics, or migraine diagnostic criteria (Figure 2).

This forest plot displays the individual and pooled trigger rates of chocolate as a patient‐reported trigger. Each horizontal line represents an individual study’s proportion and its 95% CI, while the diamond at the bottom represents the pooled estimate of 13.9% (95% CI: 0.07 to 0.21, *p* < 0.001).

#### 3.1.3. Cheese Trigger Rate

**FIGURE 3:**
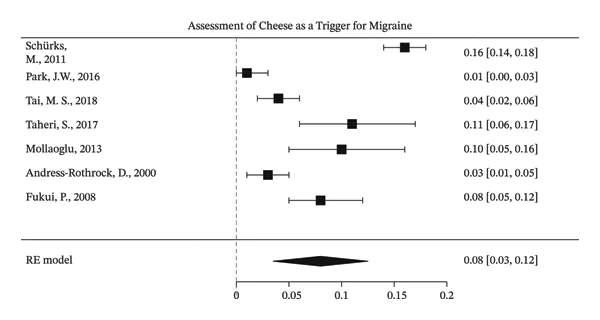
Assessment of cheese as a trigger for migraine (*n* = 7). Peris et al. were not included because extractable numerical data were unavailable.

This meta‐analysis examined the association between cheese consumption and migraine incidence across the seven included studies. The RE model was applied due to the high heterogeneity among studies (I^2^ = 95.1%), indicating substantial variation across the study results. The pooled effect size was 0.08 (95% CI: 0.03 to 0.12, *p*‐value < 0.001). This suggests a statistically significant association between cheese consumption and migraine risk, with cheese being a potential trigger (Figure 3).

This figure presents the meta‐analysis results for cheese consumption as a migraine trigger across the included studies. The pooled effect size is shown as 8% (95% CI: 0.03 to 0.12, *p* < 0.001). The plot includes the I2 statistic of 95.1%, which indicates substantial variation in results across different clinical settings and geographic locations.

#### 3.1.4. Caffeine Trigger Rate

**FIGURE 4:**
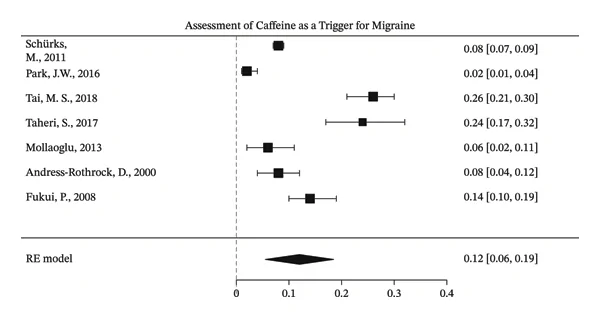
Assessment of caffeine as a trigger for migraine (*n* = 7). Peris et al. were not included because extractable numerical data were unavailable.

The association between caffeine intake and the risk of migraine has beem examined . A RE model was applied due to high heterogeneity across studies (I^2^ = 97.34%). The pooled effect size was 0.12 (95% CI: 0.06 to 0.19, *p*‐value < 0.001), suggesting that caffeine consumption may be associated with an increased risk of migraines (Figure 4). Sensitivity analysis confirmed that the significance of the pooled effect remained robust even after excluding individual studies, suggesting that the identified associations are consistent despite the high statistical heterogeneity.

This forest plot synthesizes the trigger rates for caffeine, showing a pooled rate of 12% (95% CI: 0.06 to 0.19, p<0.001). The visualization highlights the extreme heterogeneity (I2 = 97.34%).

#### 3.1.5. Risk‐of‐Bias Assessment

Four studies were rated as good and four as fair. None of the studies was rated as poor. The most common methodological limitations included reliance on self‐reported dietary triggers, inadequate adjustment for potential confounding variables, recruitment from specialist headache clinics, and limited generalizability of the study populations. Nevertheless, most studies clearly defined their research objectives, study populations, exposures, and outcomes (Table 2).

**TABLE 2:** Quality assessment of included studies using the NHLBI quality assessment tool.

Study	Overall quality	Main methodological limitations
Fukui PT et al., 2008	Fair	Self‐reported dietary triggers; limited adjustment for confounders
Andress‐Rothrock D et al., 2010	Fair	Selection from headache clinic; recall bias
Schürks M et al., 2011	Good	Limited generalizability (female health professionals only)
Mollaoğlu M., 2013	Fair	Convenience sampling; limited confounder adjustment
Park JW et al., 2016	Good	Self‐reported exposure assessment
Taheri S., 2017	Fair	Small sample size; recall bias
Peris F et al., 2017	Good	Limited adjustment for potential confounders
Tai ML et al., 2018	Good	Self‐reported dietary exposures

## 4. Discussion

This systematic review and meta‐analysis aimed to assess the trigger rates of chocolate, caffeine, and cheese for migraine. The included studies highlight that migraine is a multifactorial disorder, and that identifying individual triggers remains an important component of clinical management; however, triggers appear to be highly individualized rather than universally applicable.

The included studies found that most migraine sufferers experience multiple types of triggers, including nutritional ones. This was in agreement with several studies in the literature. For instance, a review reported by Sun‐Edelstein and Mauskop found that some patients might be sensitive to tyramine and caffeine. These items can trigger migraines in susceptible individuals, although such avoidance should be considered on a case‐by‐case basis rather than as a universal recommendation [30]. Another systematic review published in 2020 found that caffeine was one of the most common diet‐related triggers associated with increased frequency of migraine attacks [14].

The included studies highlight that the rate and impact of specific migraine triggers vary considerably between individuals[23, 27, 28]. This variability is reflected in the high heterogeneity observed across studies, likely due to differences in individual susceptibility, exposure levels, and study populations. Chocolate was identified as a potential trigger in approximately 14% of cases, while caffeine was associated with migraines in about 12% (I^2^ = 97.34%). Cheese showed a lower overall trigger rate of around 8%, but with substantial heterogeneity (I^2^ = 95.1%). Findings also differed by population. In a pediatric study, cheese was reported as a trigger in 13% of 100 children, and caffeine triggered headaches in 28% of children. Similarly, chocolate was reported as a trigger by 22% of children in another study [29]. In contrast, studies in adult populations did not consistently identify cheese as a significant trigger at the population level [23]. However, coffee was reported as the most common dietary trigger in one adult study, affecting 19.9% of patients with migraine and tension‐type headaches [24].

The association between migraines and the consumption of chocolate, cheese, and caffeine may be explained by several complex biochemical pathways involving vascular regulation and neurotransmitter activity. Chocolate contains compounds such as phenylethylamine and methylxanthines, which may influence serotonergic signaling and vascular tone. Given the central role of serotonin in migraine pathophysiology, fluctuations in serotonergic activity may contribute to activation of the trigeminovascular system, leading to migraine pain [31].

Furthermore, many aged cheeses are high in tyramine, a sympathomimetic amino acid formed by the breakdown of tyrosine. Tyramine may promote the release of catecholamines such as norepinephrine, resulting in initial vasoconstriction followed by rebound vasodilation, which may contribute to migraine in susceptible individuals. However, individual metabolic differences may influence sensitivity to tyramine [32].

Moreover, caffeine has a “double‐edged” role due to its effect on adenosine receptors. While acute doses can cause vasoconstriction, aiding pain relief, chronic or fluctuating intake may lead to adenosine receptor upregulation. Subsequent caffeine withdrawal or fluctuations in intake can trigger rebound vasodilation and neural hyperexcitability, precipitating an attack. In addition, caffeine influences multiple neurotransmitter systems, including adenosine and dopamine, which are involved in pain modulation and central nervous system excitability [33].

It is worth noting that what triggers a migraine attack in one person may not affect another [25, 28, 29]. Therefore, migraine triggers should be interpreted at the individual level rather than extrapolated directly from population‐level findings. It is also important to note that some factors may be premonitory symptoms rather than true triggers. Premonitory symptoms are warning signs that a migraine is coming, while triggers are factors that directly cause a migraine attack [23, 29].

Although the included studies did not extensively discuss the mechanisms behind caffeine’s role as a trigger, some research about caffeine has found that it initially constricts blood vessels but can later lead to rebound vasodilation, triggering migraines in susceptible individuals [34]. Caffeine also interacts with neurotransmitter systems such as adenosine and dopamine, contributing to its complex effects on migraine pathophysiology.

Regular caffeine consumption may lead to dependence, and abrupt withdrawal can trigger headaches, including migraines. Similarly, chocolate contains compounds such as caffeine and theobromine that may influence vascular and neurotransmitter pathways [35]. Moreover, regarding the role of cheese in triggering migraine, some cheeses contain tyramine, which is a naturally occurring compound that can cause blood vessel constriction and dilation, potentially triggering migraines in susceptible individuals [31]. Other bioactive compounds in cheese may also interact with the nervous system, although the evidence remains limited.

Some studies suggest that dietary modifications may reduce migraine frequency and severity; however, the effectiveness of such interventions depends on the individual’s trigger profile. Therefore, dietary interventions should be personalized rather than universally applied [36]. Overly restrictive dietary exclusions may unnecessarily limit dietary diversity and negatively impact quality of life without clear additional benefit [37, 38].

### 4.1. Limitations

This review included several methodological limitations that affected the reliability of its findings. Many studies relied on self‐reported data, which is prone to recall bias and may cause patients to misremember the timing or presence of specific triggers. This might lead to an overestimation of familiar triggers, while underestimating others. The complexity of individual trigger profiles further complicated the analysis, as each patient’s sensitivity to triggers varied, making it difficult to generalize findings at a population level. Additionally, distinguishing true triggers from premonitory symptoms, such as early signs of an oncoming migraine, remains challenging, and misclassification can result in misleading conclusions about what directly initiates a migraine. Furthermore, the lack of robust prospective studies, limited sample sizes, and variability in trigger definitions across studies limit the ability to establish definitive causal relationships.

### 4.2. Sources of Heterogeneity and Sensitivity Analysis

The meta‐analysis revealed substantial heterogeneity across all dietary triggers (I2 for chocolate, cheese, and caffeine exceeded 95%). This high variance stems from significant methodological differences between the included studies, such as the use of real‐time smartphone diaries versus retrospective paper‐based questionnaires, which are prone to recall bias. Furthermore, clinical diversity in the study populations, ranging from a pediatric cohort with a mean age of 10.5 years to an adult female health professional cohort, contributes to the wide range of reported trigger rates.

Sensitivity analysis using a leave‐one‐out approach indicated that the exclusion of larger population‐based studies, such as Schürks et al. (2011), slightly reduced the pooled rates but did not eliminate the overall heterogeneity. These findings suggest that while chocolate, caffeine, and cheese are statistically significant triggers at a population level (*p* < 0.001), their impact is highly individualized and influenced by geographical and demographic factors.

## 5. Conclusion

In conclusion, this systematic review and meta‐analysis highlights that chocolate, caffeine, and cheese are commonly reported migraine triggers. However, the results must be interpreted with significant caution due to the extreme heterogeneity (I2 > 95%) and the risk of bias present in the observational data. The findings underscore that migraine triggers are highly variable and subjective. Consequently, clinicians should emphasize individualized management, such as the use of headache diaries, to identify specific dietary sensitivities rather than recommending broad, restrictive diets for all patients. Further high‐quality, prospective research is necessary to move beyond patient‐reported associations toward establishing definitive causal relationships.

## Funding

No funding was received for this manuscript.

## Conflicts of Interest

The authors declare no conflicts of interest.

## Data Availability

Data sharing is not applicable to this article as no datasets were generated or analyzed during the current study.
